# Integrative proteo-transcriptomic and immunophenotyping signatures of HIV-1 elite control phenotype: A cross-talk between glycolysis and HIF signaling

**DOI:** 10.1016/j.isci.2021.103607

**Published:** 2021-12-10

**Authors:** Sara Svensson Akusjärvi, Anoop T. Ambikan, Shuba Krishnan, Soham Gupta, Maike Sperk, Ákos Végvári, Flora Mikaeloff, Katie Healy, Jan Vesterbacka, Piotr Nowak, Anders Sönnerborg, Ujjwal Neogi

**Affiliations:** 1 Division of Clinical Microbiology, Department of Laboratory Medicine, Karolinska Institutet, ANA Futura, Campus Flemingsberg, 141 52 Stockholm, Sweden; 2 Division of Chemistry I, Department of Medical Biochemistry and Biophysics, Karolinska Institutet, Campus Solna, 171 65 Stockholm, Sweden; 3 Division of Oral Diagnostics and Rehabilitation, Department of Dental Medicine, Karolinska Institutet, ANA Futura, Campus Flemingsberg, 141 52 Stockholm, Sweden; 4 Department of Medicine Huddinge, Division of Infectious Disease, Karolinska Institutet, I73, Karolinska University Hospital, 141 86 Stockholm, Sweden; 5 Manipal Institute of Virology (MIV), Manipal Academy of Higher Education, Manipal, Karnataka, India

**Keywords:** Glycobiology, Molecular biology, Immunology, Virology, Omics

## Abstract

Natural control of HIV-1 is a characteristic of <1% of HIV-1-infected individuals, so called elite controllers (EC). In this study, we sought to identify signaling pathways associated with the EC phenotype using integrative proteo-transcriptomic analysis and immunophenotyping. We found HIF signaling and glycolysis as specific traits of the EC phenotype together with dysregulation of HIF target gene transcription. A higher proportion of HIF-1α and HIF-1β in the nuclei of CD4^+^ and CD8^+^ T cells in the male EC were observed, indicating a potential increased activation of the HIF signaling pathway. Furthermore, intracellular glucose levels were elevated in EC even as the surface expression of the metabolite transporters Glut1 and MCT-1 were decreased on lymphocytes indicative of unique metabolic uptake and flux profile. Combined, our data show that glycolytic modulation and altered HIF signaling is a unique feature of the male EC phenotype that may contribute to natural control of HIV-1.

## Highlights

• Proteo-transcriptomic integration identifying features of EC phenotype
• Sex-specific differences in EC phenotypes
• Enrichment of glycolysis and HIF signaling, a unique feature in the male EC
• Enrichment of HIF signaling independent on HIF-1α protein levels in EC

## Graphical abstract

**Figure:**
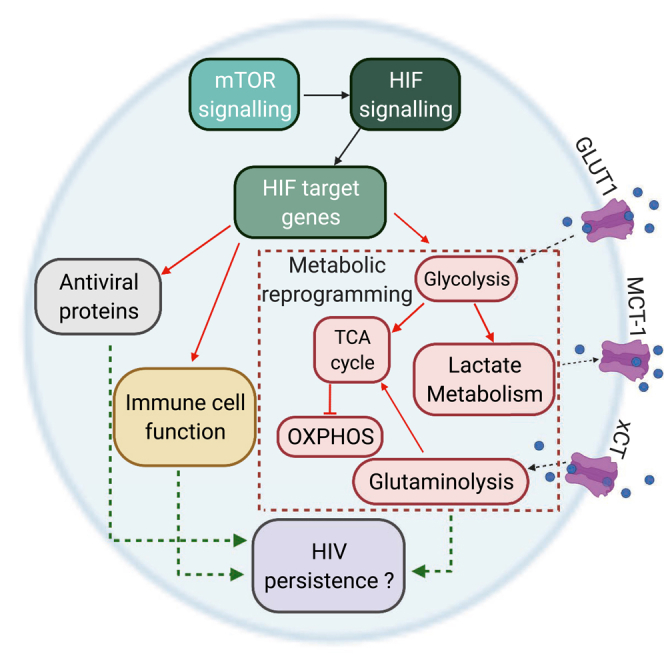

## Introduction

Elite controllers (EC) represent a small number of human immunodeficiency virus type-1 (HIV-1)-infected individuals who naturally control the infection in the absence of antiretroviral therapy (ART). The EC phenotype is rare and found in less than 1% of all HIV-1-positive individuals (Olson et al., 2014). Researchers have long strived to use the EC phenotype as a model for immunity to resolve HIV-1 disease progression with the aimed outcome of a functional cure. However, the EC phenotype has demonstrated a higher complexity than initially thought due to population-based heterogeneity while eliciting viral control. This heterogeneity is considered to be caused by variability in the host response against the pathogen as well as viral genetic elements and the location at which they integrate into the host chromosome. Immunological factors include cellular and humoral immune responses (e.g. CD8^+^ cytotoxic T lymphocytes, CD4^+^ T cell responses, NK cells, and neutralizing antibodies) while viral attenuation can be induced by deleterious mutations in the genetic material of the virus, resulting in lowered fitness and replication capacity (Poropatich and Sullivan, 2011). It has been shown that the EC phenotype is also determined by host genetic elements such as acquired immunodeficiency syndrome (AIDS) restriction genes (ARGs), exemplified by the Δ32 deletion in the CCR5 gene, and specific expression of HLA class I alleles (e.g., B57:03, B81:01), both of which are associated with protection against the infection (International et al., 2010; McLaren et al., 2012). A recent publication highlighted the importance of the HIV-1 integration site where it was shown that the majority of integrated provirus in EC was found in chromatin regions carrying repressive histone modifications (Jiang et al., 2020). This indicates that host transcriptomic regulation may be a contributing factor to the EC phenotype.

Heterogeneity in the EC phenotype has been observed between the sexes where a more distinct transcriptomic profile has been detected in male EC compared with female EC relative to their HC counterparts (Zhang et al., 2018). In addition, recent studies have focused on how the cellular immunometabolic profile regulates susceptibility to infection. As immune cell activity is determined by metabolic processes, immunometabolism can determine the response and function of cells upon pathogen encounter. In this field, a low level of inflammation and physiological oxidative stress have been proposed as contributing factors to the mechanism of EC control (Sáez-Cirión and Sereti, 2020; Sperk et al., 2021). Thus, while the EC phenotype may be regulated through an interplay of these factors, the complete mechanisms underlying natural control of HIV-1 infection remain unclear.

In this study, we conducted separate untargeted quantitative proteomic and genome-wide transcriptomic analysis to identify unique features of the EC phenotype. Furthermore, we employed integrated proteomic and transcriptomic analysis in male EC to stratify the significance of identified pathways on a complex level. The identified pathways were validated in our male and female EC cohorts to evaluate the heterogeneity between the sexes and its role in mediating the EC phenotype.

## Results

### HIF signaling and glycolysis are unique characteristics in male EC on a proteomic level

**Figure 1:**
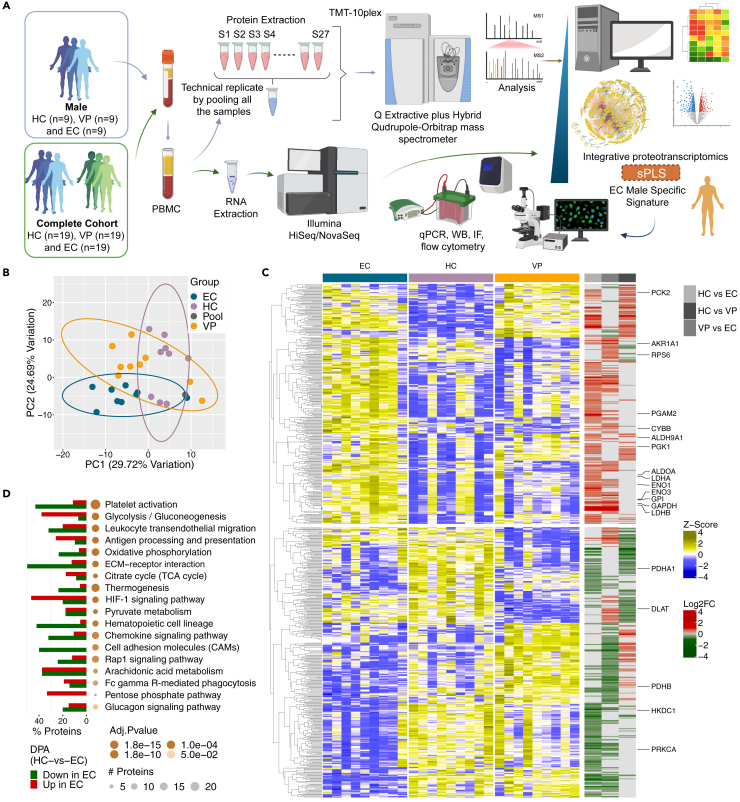
HIF signaling and glycolysis are unique characteristics in male EC on a proteomic level Study design and proteomic analysis. (A) Study design and workflow of sample processing, data generation, and integrative analysis of proteomic and transcriptomic data. PBMCs were prepared from a cohort of male HIV-1-negative individuals (HC, n = *9*), elite controllers (EC, n = *9*), and viremic progressors (VP, n = *9*); and protein lysates were analyzed on Q Exactive Plus Hybrid Quadrupole-Orbitrap mass spectrometer while RNA was sent for Illumina HiSeq/NovaSeq sequencing together with a larger cohort of both male and female HC (n = *19*), EC (n = *19*), and VP (n = *19*). Raw data processing, analysis, and data integration were performed by an in-house R script and sparse partial least squares (sPLS) regression and classification. (B) Principal component analysis showing distribution of all the samples with respect to proteomics data. The data is plotted on 2D space after normalization and batch correction. The first two principal components capturing maximum of variances were used. Data ellipses were drawn at the level of 0.95. (C) Heatmap visualizing quantile normalized and Z-scaled expression patterns of proteins significantly dysregulated in any of the pairwise comparisons among the study cohorts. Column annotations represent the cohorts and the different analysis pairs used for comparison. Proteins are clustered hierarchically based on the Euclidean distance. (D) Visualization of functional analysis of significantly dysregulated proteins in EC compared to HC. The size of the bubbles is relative to the number of features within each pathway while the color gradients represent adjusted p value of the enrichment test. The bar graph denotes number of proteins increased or decreased in each pathway. See also Figure S1.

Individual omics level analysis was first performed on transcriptomic and proteomic datasets separately to identify features associated with the EC phenotype. Previously, we have shown a strong sex bias among EC and major differences in the gene expression in male EC compared to HIV-1-negative individuals (HC) (Zhang et al., 2018). Therefore, we first focused on male EC (n = *9*), viral progressors (VP) (n = *9*), and HC (n = *9*) (Figure 1A). The normalized quantitative proteomic data were first subjected to batch correction using an inter-batch pool sample as a reference that was included in each of the three runs to assess the batchwise effect. Through batch correction of the proteomic data, the male cohort could be differentiated using principal component analysis (PCA) with adequate distinction of each group (Figure 1B). Differential proteomic analysis identified 439, 370, and 277 proteins exhibiting significant differential regulation between male EC and HC, EC and VP, and HC and VP, respectively. The high number of differential expressed proteins indicates an inter-group variation on the proteomic level, as observed in the PCA (Figure 1B). From the proteomic detection range, two clusters as shown in the heatmap distinguished the EC group from VP and HC with higher or lower levels in EC, respectively. In the VP group, smaller clusters in the heatmap were identified as dysregulated compared to HC, but the expression profile did not correlate to EC (Figure 1C). Functional analysis of significantly regulated proteins in EC compared to HC identified several pathways related to metabolism and cell signaling as enriched (Figure S1A). These enriched pathways included glycolysis (13 proteins, adjusted p < 0.001), hypoxia-inducible factor (HIF) signaling (10 proteins, adjusted p < 0.001), and the pentose phosphate pathway (PPP) (4 proteins, adjusted p = 0.023), with a higher proportion of increased proteins while platelet activation (24 proteins, adjusted p < 0.001), extracellular matrix (ECM)-receptor interaction (10 proteins, adjusted p < 0.001), hematopoietic cell lineage (9 proteins, adjusted p = 0.002), cell adhesion molecules (CAMs) pathways (10 proteins, adjusted p = 0.009), and oxidative phosphorylation (OXPHOS) (14 proteins, adjusted p < 0.001) had more decreased proteins in EC (Figure 1D).

From the transcriptomic data, PCA showed a comparative similarity between the EC and HC samples, while the VP samples did not cluster together with the other groups indicating that the VP transcriptome profile is unique in males (Figure S1B). Differential gene analysis identified 60, 3070, and 4189 genes as significantly dysregulated between EC and HC, EC and VP, and HC and VP, respectively (Figure S1C). Overall, our data indicate that HIF signaling and metabolic pathways, such as glycolysis and PPP, distinguish the male EC phenotype compared to VP and HC at a proteomic level.

### Proteo-transcriptomic integration confirms HIF signaling and glycolysis as unique features of the male EC phenotype

**Figure 2:**
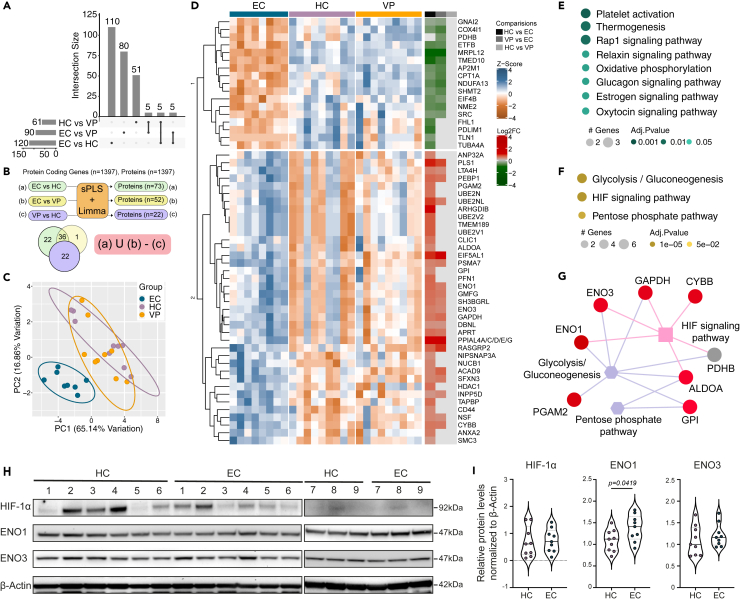
Proteo-transcriptomic integration confirms HIF signaling and glycolysis as unique features of the male EC phenotype Proteo-transcriptomic analysis in HC (n = *9*), EC (n = *9*), and VP (n = *9*). (A) Upset plot describing the number of features detected by each of the sparse partial least squares models. (B) Schematic representation of the derivation of EC-specific proteins. (C) PCA plot representing sample clustering with respect to EC-specific proteins. The first two principal components capturing maximum of variance were used. Data ellipses were drawn at the level of 0.9. (D) Heatmap visualizing quantile normalized and Z-scaled expression pattern of EC-specific proteins. Column annotation represents the cohorts and the different groups used for pairwise comparison. Proteins are hierarchically clustered based on Euclidean distance. (E) Functional analysis results of proteins belonging to cluster 1 in (D). Size of the bubble and the color gradient are relative to number of features in each pathway and adjusted p value of the enrichment test, respectively. (F) Functional analysis results of the proteins belonging to cluster 2 in (D). The bubble size and color gradient are relative to number of features in each pathway and adjusted p value of the enrichment test, respectively. (G) Schematic representation of the proteins belonging to pathways in (F). Circular nodes in red represents upregulated proteins in EC, in relation to HC, and gray color denotes non-significant expression levels. (H) Western blot analysis of HIF target genes identified in (G) for male HC (n = *9*), and EC (n = *9*). (I) Violin plots representing relative protein quantification of HIF-1α, ENO1, and ENO3 normalized to β-Actin from (H) using unpaired t test (significance level, p < 0.05) represented with violin plot with mean. See also Figure S2.

Identification of biological functions is often dependent upon signaling cascades and activation of targeted transcripts. Therefore, to better understand the cellular phenotype associated with EC, we integrated expressed transcript information and proteomic data from males in the three different cohorts. Three sparse partial least squares (sPLS) models comparing all pairs in the cohorts were generated. The model selected 61, 90, and 120 proteins to distinguish between HC and VP, EC and VP, and HC and EC, respectively (Figure 2A). Proteins specific for EC were derived by overlaying differential gene expression analysis over the selected proteins from the sPLS model and applying set operation procedures (Figure 2B). The derivation identified 59 EC-specific proteins. Of these, 22 proteins were exclusively expressed in EC *vs* HC and only one protein uniquely expressed in EC *vs* VP, while 36 proteins differentially expressed in both comparisons. Clustering analysis using PCA of the EC-specific proteins showed a cluster distinguishing the EC samples from the other two study groups, substantiating the strength of the feature derivation (Figure 2C). The EC-specific proteins exhibited a distinct expression pattern in the group, dividing into two separate clusters (Figure 2D). The differences in protein levels were mostly between EC and HC rather than between EC and VP. Cluster-specific functional analysis identified platelet activation (adjusted p < 0.001), thermogenesis (adjusted p < 0.01), and the Rap1 signaling pathway (adjusted p < 0.01), as top downregulated EC-specific pathways mapped by three proteins each (Figure 2E). The top upregulated EC-specific pathways were glycolysis/gluconeogenesis (6 proteins, adjusted p < 0.001), HIF signaling (4 proteins, adjusted p < 0.001), and PPP (2 proteins, adjusted p < 0.001) (Figure 2F). The mapped proteins in the upregulated pathways (enolase 1 [ENO1], enolase 3 [ENO3], glyceraldehyde 3-phosphate dehydrogenase [GAPDH], cytochrome b-245 heavy chain [CYBB], fructose-bisphosphate aldolase A [ALDOA], phosphoglycerate mutase 2 [PGAM2], pyruvate dehydrogenase beta [PDHB], and glucose-6-phosphate isomerase [GPI]) are linked through interaction within the identified pathways (Figure 2G). Proteins identified as having increased levels in EC compared to HC belonging to both HIF signaling and glycolysis were further validated by western blot in total peripheral blood mononuclear cells (PBMCs) (Figure 2H). The protein level of ENO1 was increased (p = 0.0419) while no significant difference was seen in the protein level of the isoform ENO3 in EC compared to HC (Figures 2H, 2I, S2A, and S2B).

To evaluate if a similar trend could be seen across the sexes, we also investigated the same protein levels in females. Herein, we detected an increase of ENO1 (p = 0.0022) in EC compared to HC (Figures S2C–S2E). In females, we also detected a decrease in protein levels of HIF-1α (p = 0.0087) in total PBMCs, which was not seen in males (Figures 2I and S2E). However, further validation of HIF-1α levels by flow cytometry did not detect any difference in either sex in PBMCs, measured by median fluorescence intensity (MFI) (Figures S2F and S2G). Therefore, these results suggest that increase in proteins associated with HIF signaling and glycolysis, independent of HIF-1α levels in total PBMCs are characteristics of the male EC phenotype.

### Dysregulation of HIF target gene transcription in EC

**Figure 3:**
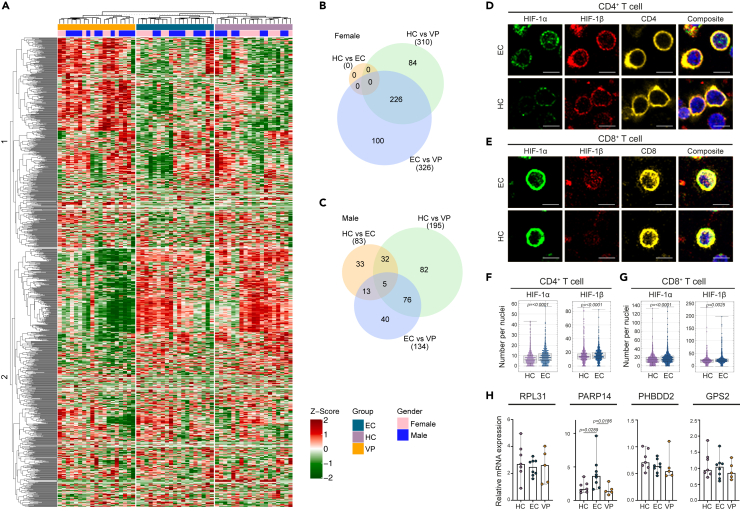
Dysregulation of HIF target gene transcription in EC Analysis of HIF target genes transcription. (A) Heatmap visualizing log2 transformed and Z-scaled expression pattern of HIF target genes from transcriptomic data of male and female HC (n = *19*), EC (n = *19*), and VP (n = *19*). Column annotation represents the study cohorts and sex of each sample. Genes are hierarchically clustered based on Euclidean distance. (B) Venn diagram of differentially expressed HIF target genes within the female cohort. (C) Venn diagram of differentially expressed HIF target genes within the male cohort. (D and E) Immunofluorescence detection of HIF-1α and HIF-1β in CD4^+^ T cells (D) and CD8^+^ T cells (E) of EC (n = 3) and HC (n = 3). Scale bar is 5μM. (F and G) Protein detection of HIF-1α and HIF-1β in nuclei of CD4^+^ (F) and CD8^+^ T cells (G). (H) qPCR validation of HIF target gene mRNA expression in male HC (*n= 7*), EC (*n= 8*), and VP (*n= 5*) of *RPL31*, *PARP14*, *RHBDD2*, and *GPS2*. (F–H) Statistical analysis was performed using Mann-Whitney U-test (significance level, p < 0.05), represented as median with 95% CI (H) or median with IQR (F and G). See also Figure S3.

As we detected HIF signaling as enriched in male EC but no difference in HIF-1α protein levels, we next looked into what effects could be detected on transcriptional activation of HIF target genes. Therefore, transcriptomic data for our complete cohort of EC (n = *19*), in addition to VP (n = *19*) and HC (n = *19*), were used to study the expression profile of HIF target genes. As we performed bulk transcriptomics on the PBMCs, we first used the EPIC to estimate the proportions of the different cell types from the bulk gene expression data (Racle and Gfeller, 2020). The largest fraction came from CD4^+^ and CD8^+^ T cells and there were no significant difference between HC and EC (p = 0.36) but as expected VP had lower CD4 T cell count (Figure S3A). We created a gene set of HIF targeted genes (n =*1288*) extracted from the Harmonizome database. The hierarchical clustering analysis of all genes showed a distinct expression profile in the EC samples, with two different clusters (Figure 3A). HIF target genes that had a lower expression in EC compared to HC included *CXCR4*, *RHBDD2*, SNRPC, and *EIF5A*, while *SLC15A2* and *GPS2* had a higher expression (Figure S3B). On a sex-specific level, no differentially expressed genes (DEG) belonging to the HIF target genes were detected between female EC and HC (Figure 3B). In females, 310 DEG were detected between VP and HC, and 326 DEG between EC and VP with an overlap of 226 DEG. Sex-specific differential expression analysis in the male cohort identified 83 differentially expressed HIF target genes between EC and HC, where 33 DEG were specifically identified between EC and HC (Figure 3C). The males also exhibited fewer DEG between VP *vs* HC (195 DEG) and EC *vs* VP (134 DEG) with less overlap between the two groups (76 DEG). From this data, the heterogeneity in the EC phenotype became even more evident on a sex-specific level. Furthermore, within the male cohort, 5 genes were differentially expressed between all three comparisons (EC *vs* HC, EC *vs* VP, and VP *vs* HC). These genes showed diverging expression patterns with two genes (*RPL39* [adjusted p = 0.0002] and *RPL31* [adjusted p = 0.022]) downregulated in EC and three genes (*PARP14* [adjusted p = 0.046], *PARP12* [adjusted p = 0.046], and *XAF1* [adjusted p = 0.0493]) upregulated in EC compared to HC (Figure S3C).

High immune activation can induce transcription of HIF target genes through HIF-1α and HIF1β translocation into the nucleus, resulting in modulation of metabolic pathways, innate and adaptive immune function, cytokine production, and phagocytosis (Palazon et al., 2014; Masoud and Li, 2015). As no overall difference in HIF-1α protein levels was detected in total PBMCs of male EC but there was a difference in transcriptional activation of HIF targeted genes, we sought to determine if this was caused by increased nuclear translocation of HIF-1α and HIF-1β in CD4^+^ and CD8^+^ T cells. As a proxy of activation of HIF signaling, we evaluated the localization of HIF-1α and HIF-1β proteins in CD4^+^ and CD8^+^ T cells of male EC (n = *3*) and HC (n = *3*) (Total CD4^+^ T cells analyzed; 1743 in EC, 1770 in HC; Total CD8^+^ T cells analyzed; 1361 in EC, 1122 in HC) (Figures 3D, 3E and S4A–S4C). Increased protein level was detected in the nuclei of both CD4^+^ (HIF-1α, p < 0.0001, HIF-1β, p < 0.0001) and CD8^+^ T cells (HIF-1α, p < 0.0001, HIF-1β, p < 0.0025) in EC compared to HC (Figures 3F and 3G). We further employed qPCR analysis for selected HIF downstream targets, dysregulated between EC and HC in the proteo-transcriptomic integration, such as *RPL31*, *PARP14*, *RHBDD2*, and *GPS2* for DEG validation. Within the male cohort, HC (*n= 7*), EC (*n= 8*) and VP (*n= 5*), we detected an increase in *PARP14* in EC compared to both HC (p = 0.0289) and VP (p = 0.0186) while no significant differences was detected in *RPL31*, *RHBDD2*, or *GPS2* (Figure 3H). Furthermore, to evaluate the heterogeneity between the sexes, we performed the same qPCR detection of selected HIF target genes in the female cohort of HC (n = *8*), EC (n = *8*), and VP (n = *4*). Herein, *RPL31* was exclusively overexpressed in VP compared to both HC (p = 0.0485) and EC (p = 0.0081) while *RHBDD2* (p = 0.0207) and *GPS2* (p = 0.0499) was decreased in EC compared to HC (Figure S3D). No difference was seen on *PARP14* transcription in the female cohort. In conclusion, our results suggest that there are sex-specific dysregulation of HIF targeted genes and a higher proportion of HIF-1α and HIF-1β is localized in the nuclei of CD4^+^ and CD8^+^ T lymphocytes in male EC under normoxic conditions.

### The metabolic uptake and secretion profiles are unique in EC

**Figure 4:**
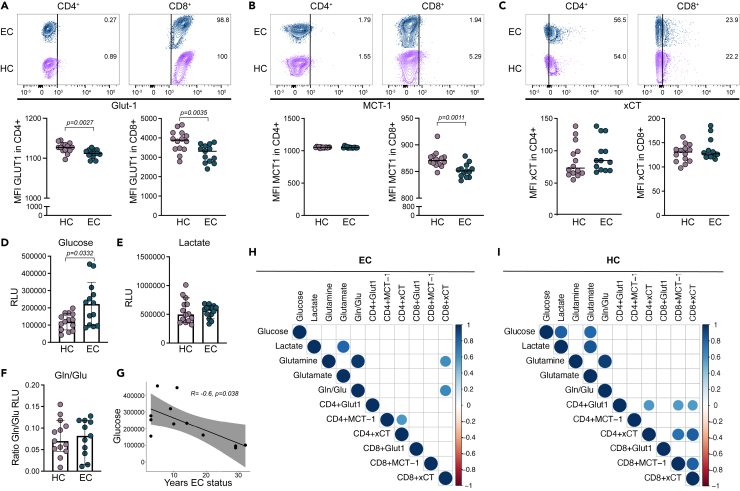
The metabolic uptake and secretion profiles are unique in EC Metabolite secretion and uptake analysis in EC (n = *13*) and HC (n = *14*). (A–C) Receptor expression on CD4^+^ and CD8^+^ T cells of Glut-1 (A), MCT-1 (B), and xCT (C). Contour plots are representative images showing median expression profile in % of cells and graphs show the median fluorescence intensity (MFI). (D–F) Intracellular levels of glucose (D), lactate (E), and glutamine/glutamate (Gln/Glu) ratio (F). Graphs show the relative light unit (RLU) and were performed in duplicates. (G) Correlation analysis of intracellular glucose levels with duration of EC status. (H and I) Correlation matrix of intracellular metabolite levels and transporter expression in EC (H) and HC (I). Statistical analysis was performed using Mann-Whitney U-test (significance level, p < 0.05) and represented with median and 95% CI or Spearman correlation (significance level, p < 0.05). See also Figure S4.

An enrichment of the glycolytic pathway was a feature detected in our male EC cohort and glycolytic genes that are mostly under the transcriptional control of HIF-1α (Kierans and Taylor, 2021). Therefore, to evaluate the metabolic uptake and secretion profiles, we measured intracellular metabolite levels of glucose, lactate, glutamine, and glutamate in total PBMCs with their corresponding transporter surface expression on lymphocytes (CD4^+^, CD8^+^) and monocytes (classical [CM], intermediate [IM], and non-classical [NCM]) in both male and female EC (n = *13*) and HC (n = *14*) (Figure S5A). Initial evaluation showed no differences between the sexes so the analysis was performed on the whole cohort (*data not shown*). Surface expression of glucose transporter (Glut1) was reduced in EC compared to HC on both CD4^+^ (p = 0.0027) and CD8^+^ T cells (p = 0.0035) (Figure 4A). The lactate transporter monocarboxylate transporter-1 (MCT-1) was reduced on CD8^+^ T cells (p = 0.0011) (Figure 4B), while no significant difference was seen on cysteine/glutamate antiporter (xCT) expression (Figure 4C). Within the monocytic cell populations, no significant differences were detected (Figures S5B–S5D). Even as transporter expression on the surface showed decreased Glut1 in lymphocytic cells from EC, total intracellular glucose levels were increased (p = 0.0332), as measured by an intracellular metabolite assay (Figure 4D). No significant differences were seen in lactate levels (p = 0.7203) or the glutamine/glutamate ratio (Gln/Glu ratio, p > 0.9999) (Figures 4E and 4F). No differences were observed in intracellular levels of glutamine (p = 0.7330) or glutamate (p = 0.3918) between the groups (Figures S5E and S5F). Untargeted metabolomics showed that plasma levels of glutamate (p = 0.0002) and lactate (p = 0.022) were significantly higher in EC compared to HC, but no difference in glutamine or glucose was detected (Figure S5G). Correlation analysis against clinical parameters showed a negative correlation of intracellular glucose levels with years of EC status (*rs = -0.6,* p = 0.038) (Figure 4G). Furthermore, both intracellular levels of lactate (EC, rs = 0.791, p = 0.002, HC, rs = 0.8555, p < 0.0001) and glutamine (EC, rs = 9.967, p < 0.0001, HC, rs = 0.947, p < 0.0001) were positively correlated in both EC and HC to glutamate and Gln/Glu ratio, respectively (Figures 4H and 4I). A positive correlation of intracellular levels between glucose and both lactate (rs = 0.815, p = 0.001) and glutamate (rs = 0.780, p = 0.002) was detected specifically in the HC group. Correlation analysis between metabolites and transporter surface expression on lymphocytes showed no shared correlations between both groups. EC had a unique phenotype with a positive correlation of CD4^+^xCT and CD4^+^MCT-1 (rs = 0.570, p = 0.045), and CD8^+^xCT with intracellular glutamine (rs = 0.613, p = 0.029) and Gln/Glu ratio (rs = 0.648, p = 0.020), respectively (Figure 4H). The HC group showed a positive correlation of CD4^+^Glut1 with CD4^+^xCT (rs = 0.573, p = 0.035), CD8^+^MCT-1 (rs = 0.562, p = 0.039), and CD8^+^xCT (rs = 0.562, p = 0.039), respectively, together with a positive correlation of CD4^+^xCT with CD8^+^MCT-1 (rs = 0.743, p = 0.003) and CD8^+^xCT (rs = 0.829, p < 0.001), as well as CD8^+^MCT-1 with CD8^+^xCT (rs = 0.817, p = 0.001) (Figure 4I). On the monocytic cell populations, the majority of correlations were detected between IM and transporter expression on CM and metabolites, respectively, in the EC group but not in HC (Figures S5H and S5I). Taken together, these results show that EC exhibit a unique metabolite uptake and secretion profile compared to HC, independent of sex.

### Dysregulated mTOR signaling in EC

**Figure 5:**
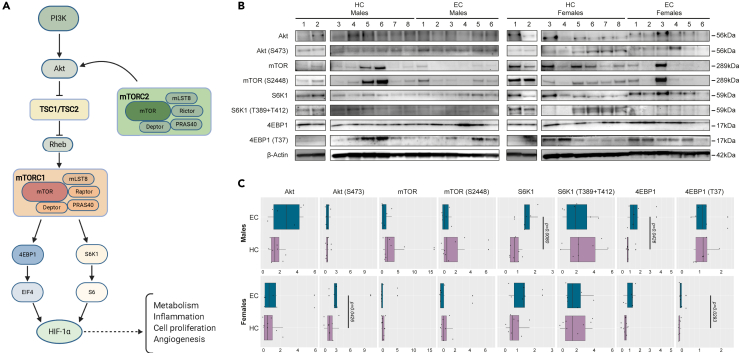
Dysregulated mTOR signaling in EC Evaluation of mammalian target of Rapamycin (mTOR) activity in male and female HC (n =16) and EC (n =12). (A) Schematic describing proteins involved in mTOR signaling. (B) Western blot image showing protein detection of Akt, Akt (S473), mTOR, mTOR (S2448), S6K1, S6K1 (T389 + T412), 4EBP1, 4EBP1 (T37), and β-Actin. (C) Relative protein quantification of Akt, Akt (S473), mTOR, mTOR (S2448), S6K1, S6K1 (T389 + T412), 4EBP1, and 4EBP1 (T37), normalized to β-Actin from (B). (C) Statistical significance was determined using Mann-Whitney U-test (Significance level, p < 0.05) and represented median with 95% CI. See also Figure S5.

Earlier studies have shown how the mammalian target of rapamycin (mTOR) pathway is upregulated in EC and a major regulator of HIV-1 latency (Chowdhury et al., 2018; Besnard et al., 2016; Martin et al., 2017). The mTOR pathway is also an upstream activator of HIF signaling and metabolic reprogramming (Figure 5A) (Luo et al., 2018). Therefore, we sought to evaluate the activity of proteins involved in mTOR signaling (Akt/Akt[S473], mTOR/mTOR[S2448], S6K1/S6K1[T389 + T412], and 4EBP1/4EBP1[T37]) in male and female EC (n = *12*) and HC (n = *16*) (Figures 5B and S6A–S6C). In the male EC, we detected elevated levels of S6K1 (p = 0.0080) and 4EBP1 (p = 0.0462), while females exhibited increased phosphorylation of Akt (S473) (p = 0.0426) and 4EBP1 (T37) (p = 0.0293) compared to respective HC (Figure 5C). This indicates that female EC have a higher activation of Akt phosphorylation not seen in the male EC.

## Discussion

In our study, elevated HIF signaling and glycolysis were detected as a signature of the male EC phenotype on a proteomic and integrated proteo-transcriptomic level. This included increased protein level of the glycolytic enzyme ENO1, earlier shown to have antiviral properties (Kishimoto et al., 2017), in combination with dysregulated expression of HIF target genes. Although HIF-1α protein levels were not increased in whole PBMCs in EC compared to HC, a higher proportion of HIF-1α and HIF-1β was localized in the nuclei of T lymphocytes in male EC, which could indicate an elevated activation of the pathway in CD4^+^ and CD8^+^ T cells. Furthermore, the EC phenotype exhibited a distinct metabolic profile with increased intracellular glucose levels and decreased Glut1 expressions on lymphocyte subpopulations, irrespective of sex. Collectively, our data show that the EC have a unique phenotype determined by a modulated HIF signaling and metabolic profile. Even as the EC phenotype is highly heterogeneous, this modulation could be one of the mechanisms contributing to natural control of HIV-1 in males.

HIF signaling is a major determinant of cellular adaptation in response to growth factors, cytokines, and infections via mTOR. Activation results in the regulation of more than 100 downstream targets mediating metabolic reprogramming together with genes promoting cell survival, proliferation, angiogenesis, and function of cellular immunity (Masoud and Li, 2015; Palazon et al., 2014). In our study, male EC showed a unique phenotype with an enrichment of HIF signaling and translocation of HIF-1α and HIF-1β into the nuclei of T cells from CD4^+^ T and CD8^+^ T lineages although no difference in total protein levels of HIF-1α. As HIF-1α is oxygen-sensitive, a rapid degradation of the protein could possibly explain our contradicting results in HIF-1α protein levels in total PBMCs or alternatively it can be attributed to insensitivity of the techniques used. Increased translocation of HIF-1α to the nuclei may result in activation of the pathway, thereby regulating transcription of HIF target genes together with T cell differentiation and function (McGettrick and O'Neill, 2020). In our study, *PARP14* were upregulated in the EC group. *PARP14* is an anti-apoptotic protein that may regulate aerobic glycolysis and differentiation of naive T cells for development of a Th2 phenotype (Iansante et al., 2015; Mehrotra et al., 2013). *PARP12* and *XAFT1* are both interferon-stimulated genes that were slightly increased in EC compared to HC while elevated in VP compared to both HC and EC which is in line with earlier reports of low level of inflammation in the EC group compared to other HIV-infected individuals (Sperk et al., 2021; Tarancon-Diez et al., 2019; Hocini et al., 2019). Contradictory, some studies have shown how the EC phenotype have a high level of inflammation, but this increased immune activation have also been attributed to be associated with loss of EC status (Noel et al., 2015; Pernas et al., 2018). The discrepancy in HIF target gene expression seen in our cohort can be attributed to the desired function for induction. As the functionality of HIF target genes ranges from metabolic reprograming, including almost all glycolytic enzymes, as well as angiogenesis, cell death mechanisms (e.g., apoptosis and autophagy), inflammation, and immunity (e.g., interferon signaling as mentioned above) together with redox homeostasis, the diverging expression levels could be what makes EC unique (Dengler et al., 2014). Stabilization of HIF-1α in normoxic conditions also inhibits HIV-1 reactivation and LTR activity (Zhuang et al., 2020). Thus, regulation of HIF signaling and its transcriptional targets indicates a role in preserving the EC phenotype by increasing the tolerance toward apoptosis and mediating an inflammatory response against HIV-1 while maintaining low level of inflammation. Furthermore, the reduced platelet activation seen in EC can be a contributing factor to the low levels of inflammation in the group. As platelet activation is one of the first line of immune system defense that can induce thrombocytopeneia, the decreased activation can be a contributing characteristic to the EC phenotype.

The second unique feature for the EC phenotype was an enrichment of proteins involved in glycolysis. Glycolytic enzymes can serve moonlighting functions by aiding in alternative cellular processes and modulating inflammatory responses (Seki and Gaultier, 2017) while also having antiviral properties (Naoki Kishimoto et al., 2012; Yu et al., 2018). In our proteo-transcriptomic integration, an upregulation of glycolytic enzymes was detected in EC compared to HC. From these enzymes, enolases are metalloenzymes that catalyze the conversion of 2-phosphoglycerate to phosphoenolpyruvate and exist in different isoforms capable of forming heterodimers. The isoform ENO1 is not one of the rate-limiting enzymes of glycolysis but it has exhibited a protective effect against HIV-1 by inhibiting the early stages of reverse transcription (Kishimoto et al., 2017, 2020; Nahui Palomino et al., 2019). Our study shows that ENO1 protein levels are increased in EC, irrespective of sex. During HIV-1 infection, these enolases may aid in the suppression of viral replication in the absence of ART. Increased levels of glycolytic enzymes also indicate a higher glycolytic flux. However, the decreased expression of Glut1 in our EC cohort could support the idea of alternative functions of these glycolytic enzymes in lymphocytes.

In terms of immune cell function, increased glycolysis is a hallmark of T cell activation. This metabolic reprogramming induces aerobic glycolysis, well-characterized from cancer cells as the Warburg effect, which upregulates Glut1 to sustain the high energetic need of bio- and macromolecules for effector function and cell proliferation (Warburg et al., 1927; Menk et al., 2018). Similar to T cell activation, earlier studies have characterized how HIV-1 infection in CD4^+^ T cells upregulates glycolysis to meet the energy-demanding turnover for virion production, simultaneously depleting CD4^+^ T cells (Hegedus et al., 2014; Palmer et al., 2014). As production of virus is an energy demanding process, it requires both increased host production of macromolecules and viral hijacking of the biosynthesis machinery (Mayer et al., 2019; Loisel-Meyer et al., 2012). During HIV-1 infection, the metabolic activity of the cell together with glycolytic enzymes and activation stage regulates susceptibility to HIV-1 where elevated OXPHOS and glycolysis favors infection in CD4^+^ T cells (Valle-Casuso et al., 2019; Clerc et al., 2019). In our cohort, we detected reduced expression of Glut1 on T lymphocytes in EC. As glucose uptake through Glut1 is a necessary process for viral production, this could potentially be a mechanism restricting propagation and infection of HIV-1 (Loisel-Meyer et al., 2012). Furthermore, intracellular glucose levels were elevated in EC compared to HC. As lymphocytes constitutes around 50% of total PBMCs, the increase in intracellular glucose levels could be a consequence of other cell types or a metabolic defect in the cells. There are also alternative glucose transporters that could mediate this increase in intracellular glucose levels, e.g., GLUT3, GLUT4, GLUT6, and SGLT1 (Lang et al., 2020). Increased intracellular glucose levels together with increased plasma levels of lactate indicate a shift toward aerobic glycolysis. In our proteo-transcriptomic analysis, we detected decreased OXPHOS in EC. This imbalance, shifting from OXPHOS could potentially be a contributing factor to the unique EC phenotype. Another feature of interest was the negative association of glucose with duration of the EC phenotype, indicating that the intracellular glucose levels normalize to HC during long-term HIV-1 infection in EC. Furthermore, as the correlation profiles of metabolites and transporter expression differed between EC and HC, this data shows that intracellular metabolite levels and their uptake and release are unique in EC group. We also observed an increase in plasma lactate levels in EC which has earlier been proposed as a stabilizer and inducer of HIF signaling (Lee et al., 2015; De Saedeleer et al., 2012; Ivashkiv, 2020; Lu et al., 2002). Increased plasma lactate levels could be a consequence of increased aerobic glycolysis or liver dysfunction which is an occurring phenomenon of untreated HIV-1 infection. In conclusion, the EC phenotype has a unique metabolite uptake and secretion profile that could reduce HIV-1 infection and replication, potentially mediated through HIF signaling, but this needs further clarification.

The heterogeneity, both in terms of mechanisms governing the EC phenotype as well as between the sexes, has previously been described (Chowdhury et al., 2018; Zhang et al., 2018) and is further strengthened by our data. Contrarily to male EC, the female EC did not exhibit increased HIF signaling but the metabolite uptake and secretion profiles did not differ between the sexes. Furthermore, the glycolytic enzyme ENO1 was increased, irrespective of sex, while elevated phosphorylation of proteins involved in mTOR signaling was seen specifically in the female EC compared to HC. Sex is an essential factor for immune cell regulation as differences occur both in responses of innate and adaptive immune cells (Klein and Flanagan, 2016). Some of these functionally different traits are specifically allocated to the X chromosome where insufficient X chromosome inactivation can yield a higher expression of certain genes in females (e.g., *TLR7* and *FOXP3*) (Libert et al., 2010). Other immune functions are highly affected by hormone levels as well as nutritional intake (Klein and Flanagan, 2016). Therefore, differential immunological functions can be attributed to the sexes and the heterogeneity of the EC phenotype. Our data thus indicates that the EC phenotype is dependent on the same metabolic reprogramming, independent of sex, but the mechanism of induction differs. To fully understand the importance of sex in the control of HIV-1 infection, further studies need to be conducted.

In conclusion, we show that glycolytic modulation together with altered HIF signaling contributes to the unique signature of the male EC phenotype. Furthermore, dysregulated HIF signaling in lymphocytic cells may result in metabolic reprogramming together with the production of antiviral proteins and modulations of immune cell functions. Together, these factors can contribute to the EC phenotype of natural control of HIV-1. Further mechanistic studies attributed to the HIF-signaling and its association with the host energy metabolism and metabolic control of the viral replication and persistence could provide insights into designing novel strategies for the functional HIV cure.

### Limitations of the study

The major limitation of this study is the small sample cohort. This is soley a consequence of the low availability of EC samples. Herein, we have included all EC available in Sweden and are thereby limited to this cohort. The technological limitations of HIF-1α detection resulted in contradicting results by western blot and flow cytometry. As the HIF-1α protein is highly oxygen-sensitive, it is possible that measurements vary depending on the technique used and the length of processing prior fixation. Furthermore, the metabolic profile we have evaluated was conducted in total PBMCs and not in single cell populations. This is a limitation as the correlation to transporter expression is performed within specific cell populations (CD4, CD8, and monocytes). Unfortunately, owing to limitations of sample availability, we were not able to conduct a more targeted metabolite measurement at the moment but in our continuing studies we are seeking to evaluate this metabolic profile in specific cellular subpopulations.

## STAR★Methods

### Key resources table

**Table:** 

REAGENT or RESOURCE	SOURCE	IDENTIFIER
**Antibodies**		

Rabbit monoclonal anti-ENO1 [EPR10863(B)]	Abcam	Cat#ab155102
Rabbit polyclonal anti-ENO3	Abcam	Cat#ab126259
Mouse IgG1, κ anti-HIF-1α (Clone 54)	BdBioscience	Cat#610959; RRID: AB_398272
Mouse monoclonal anti-HIF-1β [2B10]	Abcam	Cat#ab2771
Rabbit polyclonal anti-panAkt	Abcam	Cat#ab8805
Rabbit monoclonal anti-Akt (S473) [EP2109Y]	Abcam	Cat#ab81283
Rabbit monoclonal anti-mTOR [Y391]	Abcam	Cat#ab32028
Rabbit monoclonal anti-mTOR (S2448) [EPR426(2)]	Abcam	Cat#ab109268
Rabbit monoclonnal anti-S6K1 [E343]	Abcam	Cat#ab32529
Rabbit polyclonal anti-S6K1 (T389 + T412)	Abcam	Cat#ab60948
Rabbit monoclonal anti-4EBP1 [Y329]	Abcam	Cat#ab32024
Rabbit monoclonal anti-4EBP1 (T37) [EPR729(2)Y]	Abcam	Cat#ab75767
Mouse monoclonal anti-β-Actin (AC-15)	Sigma-Aldrich	Cat#A5441
Goat polyclonal anti-CD4	R&D systems	Cat#AF-379-NA
Rabbit monoclonal anti-CD8 Alexa Fluor 647[EP1150Y]	Abcam	Cat#ab196193
Rabbit polyclonal anti-HIF-1α	Novus Biologicals	Cat#NB-100-449
Goat polyclonal anti-rabbit Alexa Fluor 488	Invitrogen	Cat#A32731; RRID: AB_2633280
Goat polyclonal anti-mouse Alexa Fluor 568	Invitrogen	Cat#A-11004; RRID: AB_2534072
Donkey polyclonal anti-goat Alexa Fluor 647	Invitrogen	Cat#A-21447; RRID: AB_2535864
Alexa Fluor 647 anti-HIF-1α (Clone 54)	BdBiosciences	Cat#565924; RRID: AB_2739388
Fluorescein conjugated anti-Glut1 (202915)	R&D systems	Cat#FAB1418F
Alexa Fluor 594 rabbit polyclonal anti-xCT	Novus Biologicals	Cat#NB300-318AF594
Alexa Fluor 405 Monoclonal mouse anti-MCT-1 (882616)	R&D systems	Cat# FAB8275V
BV711 anti-CD3 (OKT3)	BioLegend	Cat#317306; RRID: AB_571907
BUV395 anti-CD4 (SK3)	BdBioscience	Cat#563550; RRID: AB_2738273
APC anti-CD8 (RPA-T8)	BioLegend	Cat#301014; RRID: AB_314132
BV510 anti-CD14 (M5E2)	BioLegend	Cat#301842; RRID: AB_2561946
BV786 anti-CD16 (3G8)	BdBioscience	Cat#563690; RRID: AB_2744299

**Biological samples**		

Human plasma samples	Karolinska University Hospital, Huddinge, Sweden	NA
Human PBMC samples	Karolinska University Hospital, Huddinge, Sweden	NA

**Critical commercial assays**		

Glucose-Glo	Promega	Cat#J6022
Lactate Glo	Promega	Cat#J5022
Glutamine/Glutamate Glo	Promega	Cat#J8021

**Deposited data**		

Metabolomics data	(Sperk et al., 2021)	https://doi.org/10.6084/m9.figshare.13585955.v2
Protein identification results	This manuscript:	ProteomeXchange Consortium: PXD023990
Algorithms and computer code	This manuscript	https://github.com/neogilab/ProTrans-EC
Transcriptomics	(Zhang et al., 2018)	NCBI SRA: PRJNA420459

**Oligonucleotides**		

Primers for RHBDD2, see Table S2	This paper	NA
Primer for GPS2, see Table S2	This paper	NA
Primer for RPL31, see Table S2	This paper	NA
Primer for PARP14, see Table S2	This paper	NA
Primer for Actin, see Table S2	This paper	NA

**Software and algorithms**		

Prism v8.4.3	GraphPad	https://www.graphpad.com/scientific-software/prism/
RStudio v1.4.1106	RStudio	https://www.rstudio.com
FlowJo v10.7.1	TreeStar Inc	https://www.flowjo.com
Imaris ×64 v9.6.9	Bitplane	https://imaris.oxinst.com/products/imaris-for-cell-biologists?gclid = CjwKCAiAhreNBhAYEiwAFGGKPP8onSNk1UDURAWxGzTmjFh1naZ2MlUTc3gYAiZzIybsq7HufZUnPRoC5DUQAvD_BwE
ImageLab v6.0.1	Bio-Rad Laboratories Inc	https://www.bio-rad.com/en-se/product/image-lab-software?ID=KRE6P5E8Z
R v4.1.2	R Core Team 2020	https://www.r-project.org/
Proteome Discoverer v2.2	(Orsburn, 2021)	https://www.thermofisher.com/se/en/home/industrial/mass-spectrometry/liquid-chromatography-mass-spectrometry-lc-ms/lc-ms-software/multi-omics-data-analysis/proteome-discoverer-software.html
Mascot Server search engine v2.5.1	Matrix Science Ltd	https://www.matrixscience.com
NormalyzerDE v1.12.0	(Willforss et al., 2019)	https://www.bioconductor.org/packages/release/bioc/html/NormalyzerDE.html
SVA v3.42.0	(Leek et al., 2020)	https://bioconductor.org/packages/release/bioc/html/sva.html
ggplot2 v3.3.2	(Wickham, 2009)	https://ggplot2.tidyverse.org
Limma v3.42.2	(Ritchie et al., 2015)	https://bioconductor.org/packages/release/bioc/html/limma.html
GSEAPY v0.9.16	(Subramanian et al., 2005) (Kuleshov et al., 2016)	https://pypi.org/project/gseapy/
FastQC v0.11.8	(Andrews, 2010)	https://www.bioinformatics.babraham.ac.uk/projects/fastqc/
Trim Galore v0.6.1	(Krueger, 2017)	https://github.com/FelixKrueger/TrimGalore
STAR v2.7.3a	(Dobin et al., 2013)	https://github.com/alexdobin/STAR
FeatureCounts v2.0.0	(Liao et al., 2014)	https://sourceforge.net/projects/subread/files/subread-2.0.3/
PCAtools v2.6.0	(Blighe and Lun, 2021)	https://bioconductor.org/packages/release/bioc/html/PCAtools.html
DESeq2 v1.34.0	(Love et al., 2014)	https://bioconductor.org/packages/release/bioc/html/DESeq2.html
Harmonizome database	(Rouillard et al., 2016)	https://maayanlab.cloud/Harmonizome/gene_set/HIF1A/TRANSFAC + Predicted + Transcription + Factor + Targets

**Other**		

Vibra-Cell™Ultrasonic Liquid Processor VCX 130	Sonics & Materials Inc	NA
TMT10plex™ isobaric labelling reagents	Thermofisher Scientific	Cat#90110

### Resource availability

#### Lead contact

Further information and requests for resources and reagents should be directed to and will be fulfilled by the lead contact, Ujjwal Neogi (ujjwal.neogi@ki.se). For the Elite control cohort contact should be directed to Anders Sönnerborg (anders.sonnerborg@ki.se).

#### Materials availability

This study did not generate new unique reagents.

### Experimental model and subject details

#### Patient material

The patient cohort consisted of Elite Controllers (EC, n = *19*), Viral Progressors (VP, n = *19*), and HIV-1-negative individuals (HC, n = *19*), see clinical parameters Table S1. EC characteristics were determined as HIV-1-positive for >3 years with at least 3 consecutive measurements of viral load <75 copies/mL blood. The cohort was age matched and normally distributed between the sexes. Peripheral blood mononuclear cells (PBMCs) and plasma was collected from whole blood of all individuals by density gradient centrifugation.

The research was approved by the regional Ethics Committee in Stockholm, Sweden and carried out according to the Code of Ethics of the World Medical Association (Declaration of Helsinki). All patients gave informed consent and data was anonymized and delinked before analysis.

### Method details

#### Protein preparation and chemical labelling

Cell pellets were resuspended in 40 μL of 0.1% ProteaseMax (Promega), 4 M urea (Sigma-Aldrich), 50 mM ammonium bicarbonate, and 10% acetonitrile (AcN). The samples were probe sonicated using Vibra-cell™ Ultrasonic Liquid Processor VCX 130 (Sonics & Materials, Inc.) for 1 min, with pulse 2/2, at 20% amplitude and sonicated in bath for 5 min, followed by vortexing and centrifugation for 5 min at 13,000 rpm. Protein concentrations were determined in supernatants in a 1:2 dilution in water. Protein yields varied between 3 to 95 μg. Ten μg of each sample (except for EC1 [7 μg], HC13 [3 μg], HC17 [3 μg], VP3 [8 μg], and VP10 [3 μg]) were subjected to a tryptic digestion, following protein reduction with 6 mM dithiothreitol at 37°C for 60 min and alkylation with 22 mM iodoacetamide for 30 min at room temperature (RT) in dark. Trypsin was added in an enzyme to protein ratio of 1:50 and digestion was carried out at 37°C over night. Tryptic peptides were cleaned on C18 HyperSep™ filter plate with 40 μL bed volume (ThermoFisher Scientific) and dried on a speedvac (miVac, Thermo Scientific). TMT10plex™ isobaric labelling reagents (ThermoFisher Scientific) in 100 μg aliquots were dissolved in 30 μL dry AcN, scrambled and mixed with the digested samples dissolved in 70 μL triethylammonium bicarbonate, followed by incubation at 22°C for 2 h at 550 rpm. The reaction was then quenched with 12 μL of 5% hydroxylamine at 22°C for 15 min at 550 rpm. The labelled samples were pooled and dried on a speedvac (miVac, ThermoFisher Scientific).

#### Liquid chromatography-tandem mass spectrometry

The TMT-labelled tryptic peptides were dissolved in 20 μL of 2% AcN/0.1% formic acid. Five μL samples were injected into a EASY-nLC™1000 liquid chromatography system (ThermoFisher Scientific) on-line coupled to a Q Exactive™ Plus Hybrid Quadrupole-Orbitrap™ mass spectrometer (ThermoFisher Scientific). The chromatographic separation of the peptides was achieved using a 50 cm long EASY-Spray™ C18 column (ThermoFisher Scientific), with an organic gradient: 4–26% B (solvent B = 98% AcN/0.1% formic acid) in 180 min, 26–95% B in 5 min, and 95% B for 8 min at a flow rate of 300 nL/min. The mass spectrometric (MS) acquisition method was comprised of one survey full spectrum ranging from *m/z* 350 to 1600, acquired with a resolution of R = 140,000 (at *m/z* 200), followed by data-dependent higher energy collision dissociation (HCD) fragmentations of maximum 16 most intense precursor ions with a charge state 2+ and 3+, applying 60 s dynamic exclusion. The tandem mass scans were acquired with a resolution of R = 70,000, targeting 2x10^5^ ions, setting isolation width to 2.0 Th and normalized collision energy to 33%.

#### Protein identification and quantification

The raw data files were directly loaded in Proteome Discoverer v2.2 (ThermoFisher Scientific) and searched against human SwissProt protein databases (21,008 entries) using the Mascot Server v2.5.1 search engine (Matrix Science Ltd.) (Orsburn, 2021). Parameters were chosen as follows: up to two missed cleavage sites for trypsin, precursor mass tolerance 10 ppm, and 0.05 Da for the HCD fragment ions. Dynamic modifications of oxidation on methionine, deamidation of asparagine and glutamine and acetylation of N-termini were set. For quantification both unique and razor peptides were requested. The final quantitative data analysis was performed with an in-house developed R-studio script, as described below.

#### Proteomic analysis

The raw data generated from the machine was first subjected to data normalization using the R/Bioconductor package NormalyzerDE (Willforss et al., 2019). The R package performs data normalization using eight different methods. Normalization using quantile method was found to be most appropriate and used for further downstream analysis. Batch effect was corrected using R function ComBat from the package SVA (Leek et al., 2020). Sample distribution was evaluated using dimensionality reduction by PCA with R package PCAtools. First two principal components capturing maximum variance of the data were plotted in 2D space using R package ggplot v3.3.2 (Wickham, 2009). Differentially protein abundance between groups were identified by R/Bioconductor package Limma v3.42.2 (Ritchie et al., 2015). Proteins with adjusted *p*-value < 0.05 were considered as significant. Functional analysis of significantly regulated proteins was performed using enrichr module of python package GSEAPY v0.9.16 (https://pypi.org/project/gseapy/) (Subramanian et al., 2005) (Kuleshov et al., 2016). Enrichment test for molecular pathway analysis was performed by KEGG gene-set library downloaded from Enrichr web resource. Proteomics data after normalized batch correction can be viewed in Table S2.

#### RNA sequencing (RNA-seq)

Total RNA was extracted from PBMCs and processed at the National Genomics Infrastructure, Science for Life Laboratory, Stockholm, Sweden (Zhang et al., 2018). The raw sequences were first subjected to quality check using FastQC tool kit version 0.11.8 (Andrews, 2010). Illumina adapter sequences and low-quality bases were removed from the raw reads using the tool Trim Galore version 0.6.1 (Krueger, 2017). Phred score of 30 was used as cut-off to remove low-quality bases prior quality assessment to assure high-quality data for further analysis. The pre-processed reads were aligned against human reference genome version 38 Ensembl release 96 using short read aligner STAR version 2.7.3a (Dobin et al., 2013). Gene level read count data was generated for each sample using the module featureCounts (Liao et al., 2014) from the software subread version 2.0.0. Human reference genome annotation version 38 Ensembl release 96 was used for the read counting. Sample distribution based on transcriptomics data was checked by performing dimensionality reduction using PCA with R package PCAtools (Blighe and Lun, 2021). First two principal components capturing maximum variance of the data were plotted in 2D space using R package ggplot v3.3.2 (Wickham, 2009). Differential gene expression analysis was performed using R/Bioconductor package DESeq2 (Love et al., 2014). Genes with adjusted *p*-value less than 0.05 were regarded as significantly expressed genes. HIF1A target genes were retrieved from the Harmonizome database (https://maayanlab.cloud/Harmonizome/gene_set/HIF1A/TRANSFAC+Predicted+Transcription+Factor+Targets) (Rouillard et al., 2016).

#### Proteo-transcriptomics integration

R/Bioconductor package mixOmics (Rohart et al., 2017) was used for integrating proteomics and transcriptomics data. Genes corresponding to proteins detected from the proteomics experiments were selected from the transcriptomics dataset and used for integration. The two omics data levels were merged using sPLS method in regression model. Proteomics data was used as predictor variable whereas transcriptomics data was used as response variable. Three separate sPLS models were generated for each pairs of groups among the cohorts. Number of features was optimized by visual inspection of the sample distribution.

#### EC specific proteins

Proteomics features selected by the three sPLS models were considered for extracting proteins specifically expressed in EC. First, proteins with significant expression levels were mined from the list of sPLS selected proteins. The resulting list of proteins was subjected to basic set operations. Union of proteins from the sPLS models created using the pairs EC and HC and EC and VP were created. Proteins from the sPLS model of HC and VP were then removed from the union list. The resultant list of proteins was considered as sPLS EC specific. Further significantly expressed proteins between each of the three comparisons were selected from the sPLS EC specific proteins and aforementioned set operations were again performed. The final list of proteins was regarded as EC specific proteins. Sample distribution by dimensionality reduction using EC specific proteins was used to access the robustness of the method.

#### Visualization of proteomic and transcriptomic data

Heatmaps were generated using R/Bioconductor package ComplexHeatmap v2.2.0 (Gu et al., 2016). Bubble plots and bar graphs were created using geom_point and geom_bar objects from R package ggplot2 v3.3.2, respectively. Upset plot was created using R package UpSetR. Network type visualization was made using Cytoscape.

#### Western blot

Patient PBMCs were lysed in RIPA buffer supplemented with cOmplete™ Protease Inhibitor Cocktail (Roche) and PhosSTOP (Roche) on ice. Protein concentration was determined using DC protein assay (Bio-Rad Laboratories) and samples prepared in 1x NuPage loading buffer (Invitrogen™) with 1x NuPage Sample Reducing Agent (Invitrogen™). Samples were run on NuPage Bis-Tris 4-12% (Invitrogen™) and NuPage Tris-Acetate 3–8% (Invitrogen™) gels and transferred using iBlot dry transfer system (Invitrogen™). Membranes were blocked 1h at RT using 5% bovine serum albumin or milk in PBSt 0.1% Tween-20 and primary antibody incubated over-night at 4°C, ENO1 (Abcam, #ab155102), ENO3 (Abcam, #ab126259), HIF-1α (BdBioscience, #610959), Akt (Abcam, #ab2771), Akt(S473) (Abcam, #ab81283), mTOR (Abcam, #ab32028), mTOR(S2448) (Abcam, #ab109268), S6K1 (Abcam, #ab32529), S6K1(T389 + T412) (Abcam, #ab60948), 4EBP1 (Abcam, #ab32024), 4EBP1(T37) (Abcam, #ab75767), or β-Actin (Sigma-Aldrich, #A5441). The secondary antibody (Dako, Aglient) was incubated 1h at RT prior detection using Amersham ECL/ECL select (GE Healthcare). Relative protein quantification was analysed using ImageLab version 6.0.1 (Bio-Rad Laboratories), results analysed using Mann-Whitney U-test or unpaired t-test and visualized using Prism 8.4.3 (GraphPad Software) (significance level, *p<0.05*).

#### Flow cytometry

HIF-1α protein expression in PBMCs was evaluated using the Transcription factor buffer kit (BD Bioscience) according to the manufactures protocol. Receptor expression of CD3(clone OKT3; Biolegend), CD4(clone SK3; BdBioscience), CD8(clone RPA-T8; BioLegend), CD14(clone M5E2; BioLegend), CD16(clone 3G8; BdBioscience), Glut1(clone202915; R&D systems), xCT(Novus Biologicals), and MCT-1(clone882616; Novus Biologicals) were evaluated by staining in FACS buffer (2%BSA, 1mM EDTA) at 4°C for 30 min prior fixation using 2% PFA. All staining was complemented with V500 Aqua viability stain (Invitrogen™) or Near-IR viability stain (Invitrogen™). Samples were quired on FACS Fortessa (BD Bioscience) using the 405 and 639 lasers with filers 525/50 and 670/30 for HIF-1α and on FACS Symphony (BD Bioscience) with laser and filter settings 355; 379/28, 406; 450/50, 525/50, 710/50, 810/40, 488; 530/30, 561; 610/20, and 637; 670/30, 780/60 for metabolic transporter expression. Data was analysed in FlowJo™ 10.7.1 and statistical analysis performed using Mann-Whitney U-test in Prism 8.4.3 (GraphPad Software) (significance level, *p<0.05*).

#### Immunofluorescence microscopy

PBMCs (250,000/coverslip) were added in 100μL PBS on poly-L-lysine pre-coated coverslips (BioCoat™) and left to adhere 30 min at 37°C. After attachment coverslips were washed in PBS and fixed 10 min at RT using 4% PFA. Cells were blocked using 3% BSA and antibody detection of CD4 (R&D systems, #AF-379-NA) and Alexa 647 conjugated CD8 (Abcam, #ab196193) performed prior permeabilization 10 min using 0.2% Triton x-100 and subsequent primary antibody incubation targeting HIF-1α (Novus Biologicals, #NB-100-449) and HIF-1β (Abcam, #ab2771). Secondary antibodies, Alexa Fluor 488 (Invitrogen, #A32731), Alexa Fluor 568 (Invitrogen, #A-11004), and Alexa Fluor 647 (Invitrogen, #A-21447) were incubated 1h at RT. Cells were counterstained using DAPI and mounted with Prolong Gold Antifade reagent (Thermofisher). Images were acquired using Nikon single point scanning confocal microscope with 60x/1.4 oil objective. All samples were analysed in two technical replicates and detection threshold was set using secondary antibody controls. Image analysis was performed in Imaris (Bitplane) using detection of Nucleus, Cell and Vesicles detection according to pipeline in Figures S4A and S4B. Graphical representation was performed in Imaris and statistical analysis was performed using Mann-Whitney U-test in Prism 8.4.3 (GraphPad Software) (significance level, *p<0.05*).

#### qPCR

RNA was extracted from PBMCs using TRI Reagent (Zymo Research) and Direct-zol™ RNA MiniPrep kit (Zymo research) and cDNA synthesis by the SuperScript™ IV reverse transcriptase (ThermoFisher Scientific), supplemented with dNTP Mix (ThermoFisher Scientific), Random hexamer Primer (ThermoFisher Scientific) and RNasin® Ribonuclease Inhibitor (Promega). Primers targeting *RHBDD2*, *GPS2*, *RPL31*, *PARP14*, and *Actin* were run using KAPA SYBR Fast universal master mix (Roche) on ABI7500F, according to the manufacturers protocol. Primer sequences can be found in Table S3. Gene expression analysis was performed using the ΔΔCT method and statistical significance evaluated using Mann-Whitney U-test (significance level, *p<0.05*) and visualized using Prism 8.4.3 (GraphPad Software).

#### Targeted metabolite detection

Levels of glucose, glutamine, glutamate, and lactate were detected in whole PBMCs using Glucose-Glo, Glutamine/Glutamate Glo, and Lactate Glo Assays (Promega), according to the manufacturer's protocols. One HC and two EC samples were excluded as glutamine levels were negative indicating an error in measurement of glutamate. Data was analyzed using Mann-Whitney U-test, and visualized using Prism 8.4.3 (GraphPad Software) (significance level, *p<0.05*).

#### Untargeted metabolomics

Analysis of plasma metabolites composition was performed using non-targeted MS by Metabolon Inc (North Carolina, US) in HD4 platform (Sperk et al., 2021). Levels of glucose, glutamine, glutamate, and lactate were compared for differential plasma levels between the groups. Statistical significance was determined using Mann-Whitney U-test in R (significance level, *p<0.05*).

### Quantification and statistical analysis

Normality of the data was evaluated and statistical tests was performed uning Mann-Whitney U-test (p<0.05) in Prism 8.4.3 (GraphPad Software). Data representation, dispersion and precision measures can be viewed in the figure legends. Correlation analysis was performed using Spearman test creating heatmaps in Prism 8.4.3 (GraphPad Software) (significance level, *p<0.05*). Figures for single parameter correlation and correlation matrix was performed using the ggscatter and corrplot function in RStudio. Number of samples included (exact n value) in each experiment can be viewed in the figure legends. The statistics used in the bioinformatics analysis is described in the individual method sections above.

## Data Availability

• Raw transcriptomics data have been deposited in NCBI SRA (Zhang et al., 2018). Accession numbers are listed in the key resources table. Protein identification results are deposited to the ProteomeXchange Consortium (http://proteomecentral.proteomexchange.org) via the PRIDE partner repository (PXD023990) (Perez-Riverol et al., 2019). Metabolomic data is deposited in FigShare as listed in the key resources table (Sperk et al., 2021). • All computer codes has been deposited at GitHub and is publicly available as of the date of publication. Link to access the code is in the key resources table. • Any additional information required to reanalyze the data reported in this paper is available from lead contact upon request.

## Supplemental information

Document S1. Figures S1–S6 and Tables S1 and S3

Table S2. Proteomics data after normalized batch correction, related to STAR Methods
