# Supplementary material for: Integrative proteo-transcriptomic and immunophenotyping signatures of HIV-1 elite control phenotype: A cross-talk between glycolysis and HIF signaling
Source: iScience. 2021 Dec 10;25(1):103607. doi: 10.1016/j.isci.2021.103607 (PMC8718889; doi:10.1016/j.isci.2021.103607)
Supplement: Document S1. Figures S1–S6 and Tables S1 and S3 [file mmc1.pdf]

iScience, Volume *25*

Supplemental information

Integrative proteo-transcriptomic and immunophenotyping signatures of HIV-1 elite control phenotype: A cross-talk between glycolysis and HIF signaling

Sara Svensson Akusjärvi, Anoop T. Ambikan, Shuba Krishnan, Soham Gupta, Maike Sperk, Ákos Végvári, Flora Mikaeloff, Katie Healy, Jan Vesterbacka, Piotr Nowak, Anders Sönnerborg, and Ujjwal Neogi

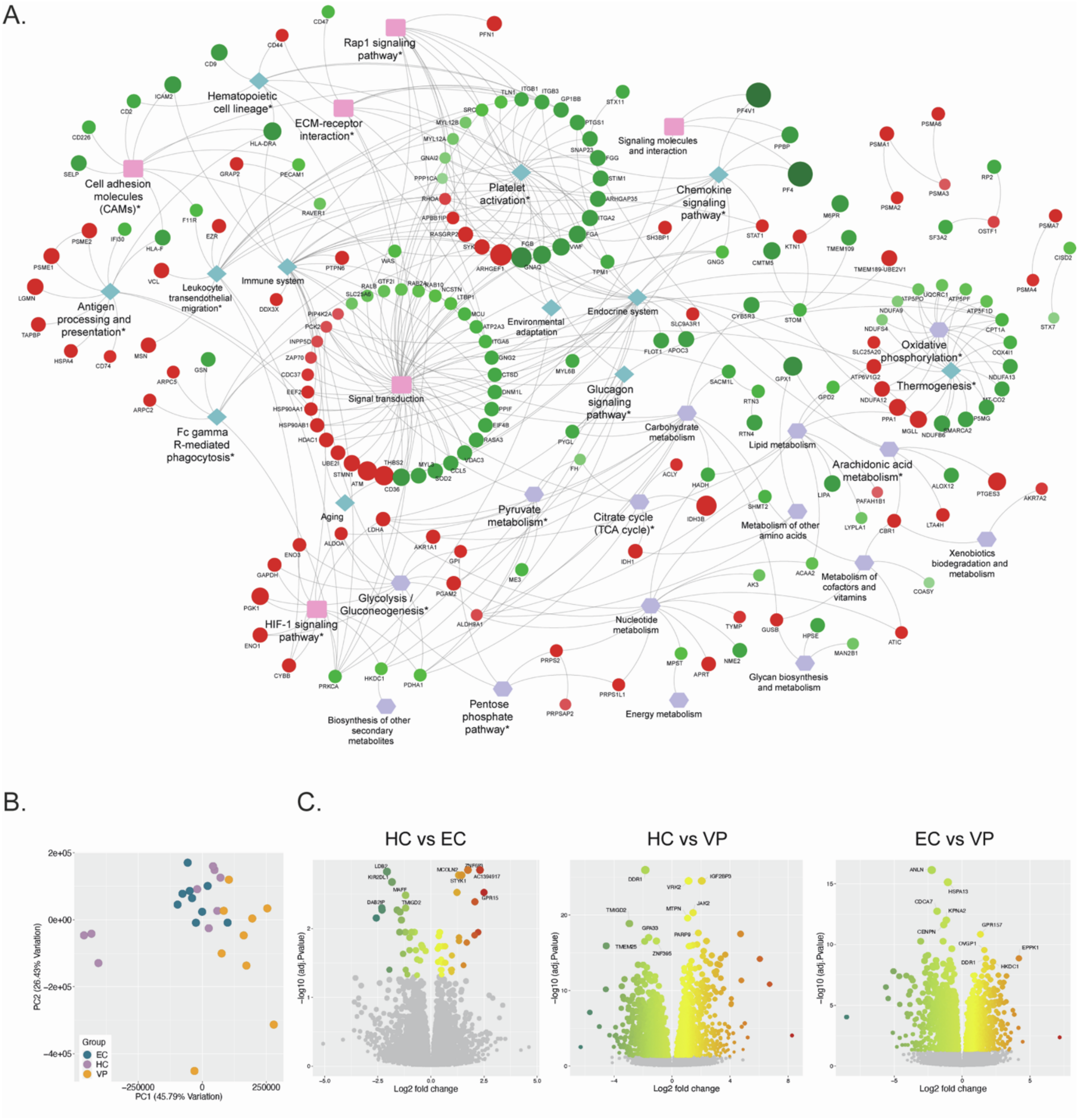

Figure S1 Identified feature of individual omics level analysis of male EC (*n*=9), VP (*n*=9) and HC (*n*=9). Related to Figure 1. (***A***) Network analysis of identified proteomic features in EC compared to HC. Red denotes significantly upregulated proteins and green denotes significantly downregulated proteins in EC from Figure 1C. (***B***) PCA plots of transcriptomic data from males. (***C***) Differential gene expression analysis of transcriptomic data in males between HC and EC, HC and VP, and EC and VP.

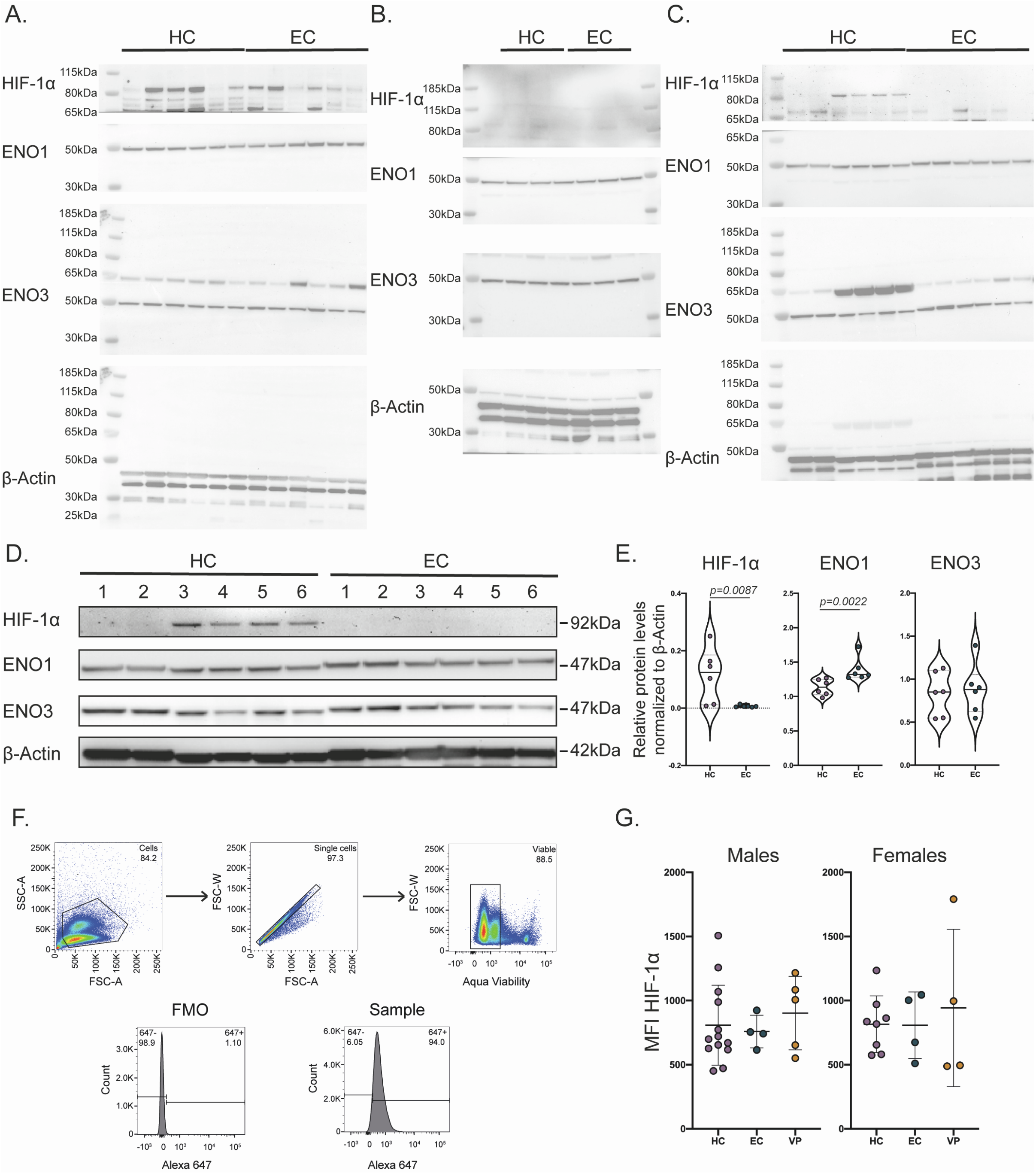

Figure S2 Protein detection of HIF-1α together with HIF target protein ENO1 and ENO3 detected as upregulated in the proteo-transcriptomic analysis in male. Related to Figure 2. (***A-C***) Raw western blot membranes for HIF-1α, ENO1, and ENO3 and β-Actin detection for male HC (*n=6*) and EC (*n=6*) (***A***), additional male HC (*n=3*) and EC (*n=3*) (***B***) and female HC (*n=6*) and EC (*n=6*) (***C***). (***D***) Western blot image showing the protein expression in HC (*n=6*) and EC (*n=6*) in females. (***E***) Relative protein quantification of HIF-1α, ENO1, and ENO3 normalized to β-Actin in females. (***F-H***) Flow cytometry analysis of HIF-1α in PBMCs of HC (*n=21*), EC (*n=8*), and VP (*n=9*). (***F***) Gating strategy showing fluorescence minus one (FMO) and sample acquisition. (***G***) Median fluorescence intensity (MFI) of HIF-1α in positive cells in males and females separately. Statistical significance was evaluated using Mann-Whitney U-test (significance level, *p<0.05*) and data represented as violin plots or median with 95% CI.

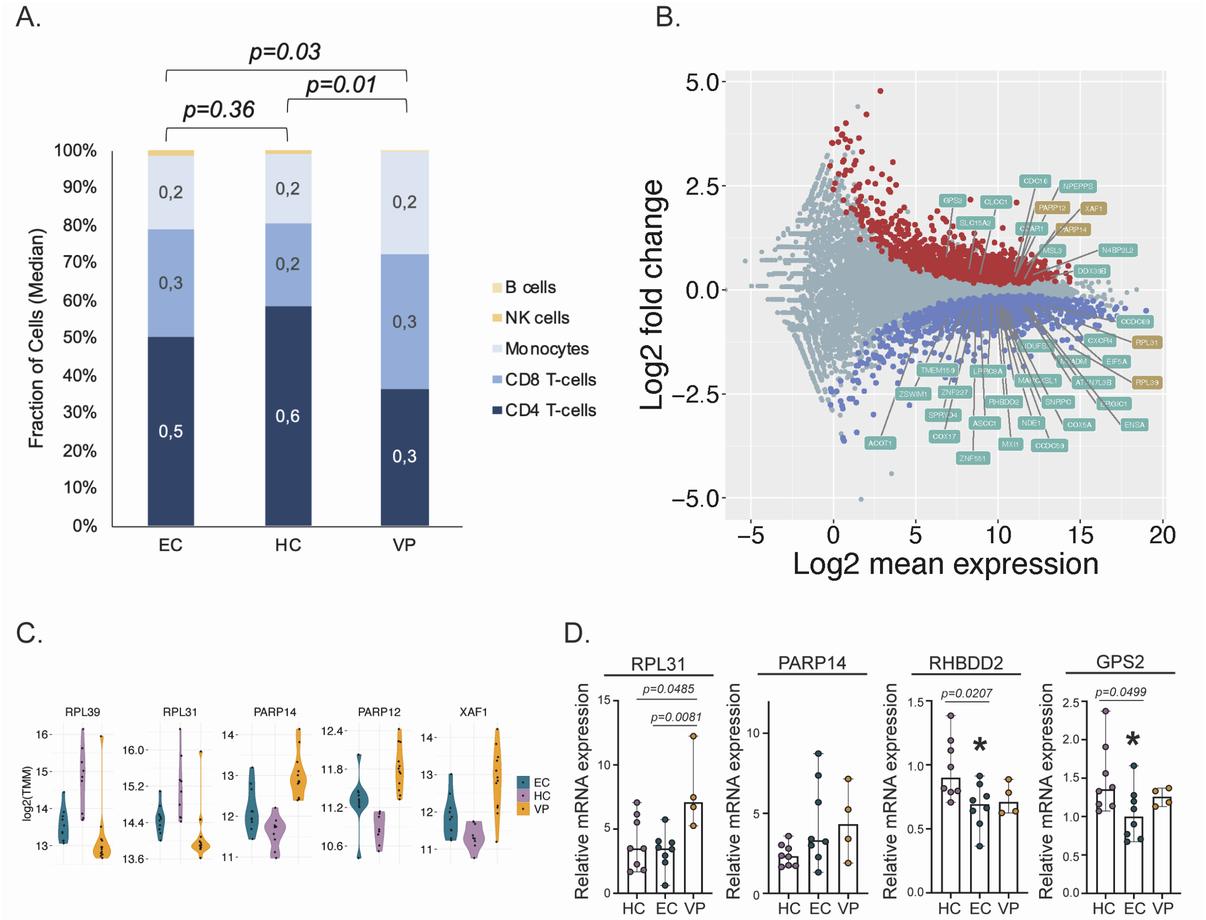

Figure S3 Detection of HIF-1 target genes. Related to Figure 3. (***A***) EPIC analysis on bulk gene expression data identifying fractions of cell types within EC (*n=9*), VP (*n=9*) and HC (*n=9*). (***B***) Differential gene expression of HIF-1 target genes in EC compared to HC. Red denotes genes that are statistically increased while blue denotes genes that are statistically decreased in EC compared to HC. (***C***) Violin plot of the significantly expressed genes between all the comparisons in Figure 3C. (***D***) Validation by qPCR in female HC (*n=8*), EC (*n=8*) and VP (*n=4*) of RPL31, PARP14, RHBDD2, and GPS2. Statistical significance was evaluated using Mann-Whitney U-test (significance level, $p<0.05$) and data represented as median with 95% CI.

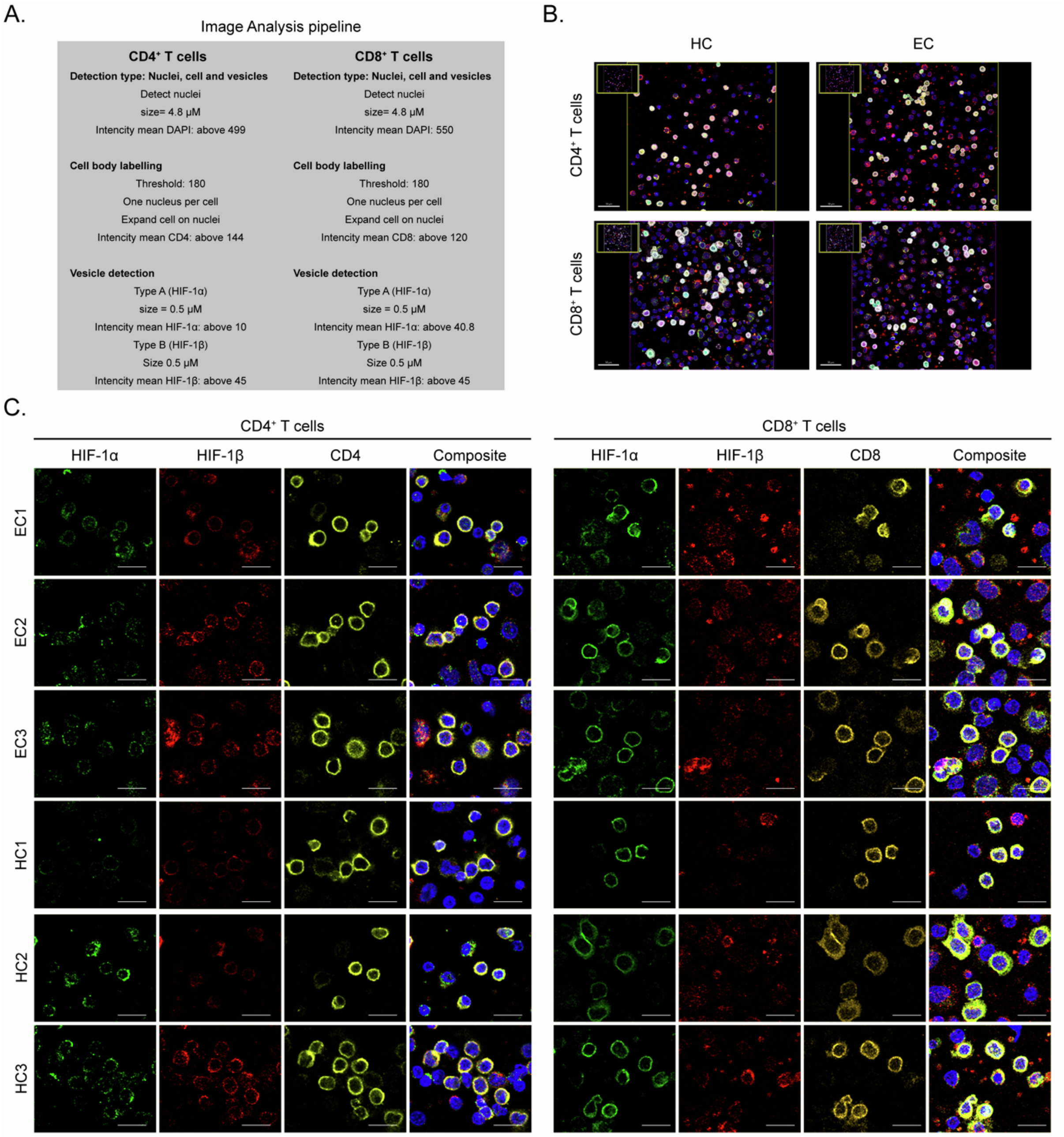

Figure S4 Immunofluorescence analysis of HIF-1α and HIF-1β localization in CD4$^+$ and CD8$^+$ T cells. Related to Figure 3. (***A***) Image analysis pipeline of CD4$^+$ and CD8$^+$ T cells expressing HIF-1α and HIF-1β in Imaris. (***B***) Representative images of HIF-1α and HIF-1β detection in CD4$^+$ and CD8$^+$ T cells of male HC (*n=3*) and EC (*n=3*) donors. Scale bar represents 30μM (***C***) Detection of HIF-1α and HIF-1β by IF in CD4$^+$ and CD8$^+$ T cells in EC (*n=3*) and HC (*n=3*) by immunofluorescence. Scale bar represents 10μM.

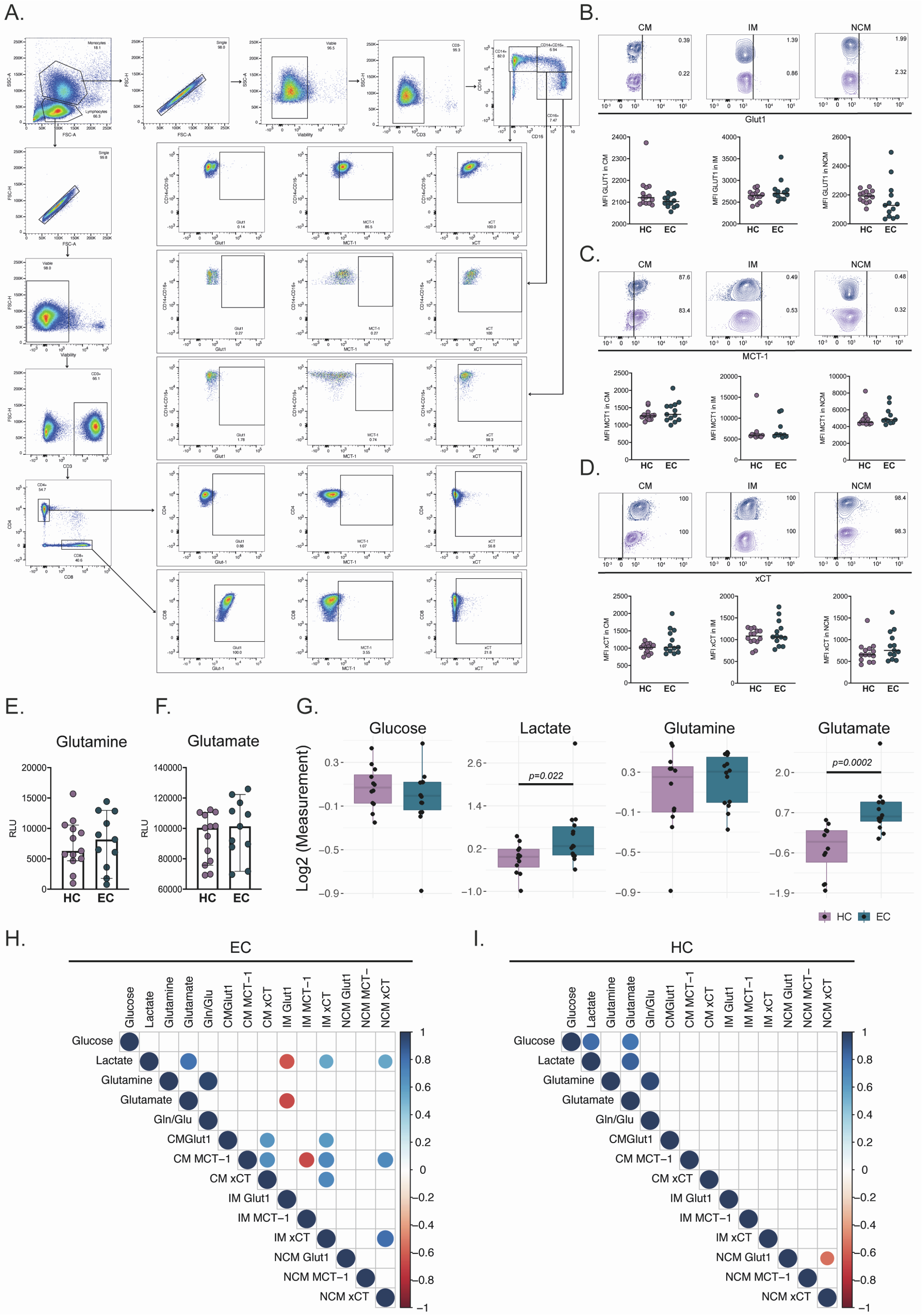

Figure S5 Metabolite uptake and release analysis in EC (*n=13*) and HC (*n=14*). Related to Figure 4. (***A***) Gating strategy of flow cytometry analysis of metabolite transporter expression

in lymphocytes ($CD4^+$, $CD8^+$) and monocytes (classical (CM), intermediate (IM), and non-classical (NCM)). (***B***) Transporter expression of Glut1 on CM, IM, and NCM. (***C***) Transporter expression of MCT-1 on CM, IM, and NCM. (***D***) Transporter expression of xCT on CM, IM, and NCM. Contour plots shows a representing image of median % of cells expressing transporters and figures show median fluorescence intensity (MFI) (***B-D***). (***E, F***) Intracellular metabolite levels of glutamine (***E***) and glutamate (***F***). (***G***) Plasma metabolites of glutamate, glutamine, lactate, and glucose in EC *(n=14*) and HC (*n=12*). (***H, I***) Correlation matrix of intracellular levels of metabolites with their corresponding transporter in EC (*n=13*) (***H***) and HC (*n=14*) (***I***). Significance was evaluated using Mann-Whitney U-test (significance level, $p<0.05$) and represented using median with 95% CI. Correlation analysis was performed using Spearman correlation (significance level, $p<0.05$).

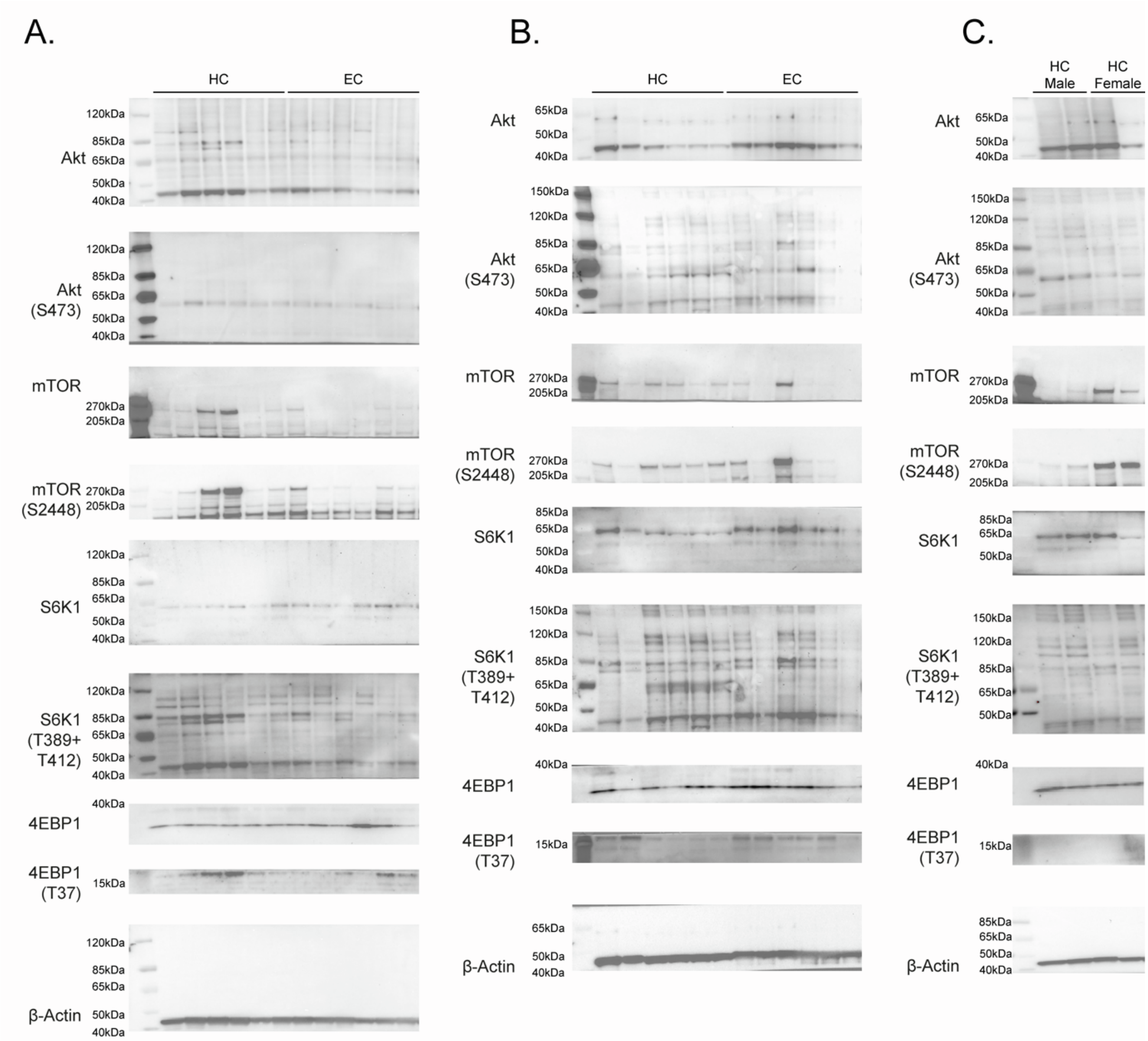

Figure S6 Raw western blot membranes for Akt, Akt (S473), mTOR, mTOR (S2448), S6K1, S6K1 (T389+T412), 4EBP1, 4EBP1 (T37), and β-Actin. Related to Figure 5. (***A, B***) Whole membranes of male HC (*n=6*) and EC (*n=6*) (***A***) and female HC (*n=6*) and EC (*n=6*) (***B***). (***C***) Additional membrane of male (*n=2*) and female (*n=2*) HC.

Table S1 Characteristics of patient cohort. Related to STAR Methods.

Parameter	EC	VP	HC	P-values
N	19	19	19	NA
Sex, Female, N (%)	9 (47.4%)	7 (36.9%)	10 (52.6%)	
**At sampling**				
Years of HIV^+ diagnosis; median (IQR)	11.7 (9)	0 (0-0)	-	<0.0001[#]
Age in years, mean (SD)	46.5 (9.8)	44.7 (13.3)	47.4 (8.5)	0.3219[*]
CD4 count (cells/µL); median (IQR)	880 (450)	330 (180)	-	<0.0001[#]
CD8 count (cells/µL); median (IQR)	660 (640)	610 (490)	-	0.9675[#]
CD4:CD8 ratio, median (IQR)	1.21 (0.77)	0.32 (0.39)	-	<0.0001[#]
Viral Load Log_{10} copies/mL (IQR)	0 (1.51)	4.89 (1.17)	-	<0.0001[#]

NA, Not Applicable; U, Unavailable; [*] One way-Anova; [#]Mann-Whitney U-test

Table S3 List of primer sequences for qPCR. Related to STAR Methods.

Target	Primer sequence
RHBDD2	F (5'-GGTGTTTGGCATGGTTGTG-3')<br>R (5'-CGATGGAATAGCAGTAGGTGA-3')
GPS2	F (5'-ACCCGCGATTCTACCACAAG-3')<br>R (5'-CTGGGACACACAGGGGATAC-3')
RPL31	F (5'-CAGAATGGCTCCCGCAAA-3')<br>R (5'-TGGCAGAACGGCCCTTT-3')
PARP14	F (5'-TGCCAAGAATGGCCAGACAA-3')<br>R (5'-GGCATAGCTGCGGTTAAAGC-3')
Actin	F (5′-GAGGGAAATCGTGCGTGACA-3′)<br>R (5′-AATAGTGATGACCTGGCCGT-3′)
